# Campaigning Notes, 1940-45

**Published:** 1946

**Authors:** Martin Herford

**Affiliations:** Rockefeller Research Fellow


### CAMPAIGNING NOTES, 1940–45

BY

COLONEL MARTIN HERFORD, D.S.O., M.B.E., M.C., M.B., CH.B., R.A.M.C.

*Rockefeller Research Fellow.*

---

TOGETHER with other Bristolians, most of whom are now out of uniform, I have been asked to record a few of my personal experiences while in the Army. Where should one begin ? The events of the last few years seem already far away. Like so many, I joined the forces and saw something of the world. Never have so many travelled so much and often, apparently, so much at random. Events crowd each other out of memory, and at times even years get mixed.

When the war started I was House Surgeon to Mr. Wright, and like many others was " fixed " at my post in expectation of heavy air raid casualties. Nothing happened. Finland was fighting, and for a variety of rather ill-defined motives, including pacifist ideas and a dislike of wearing uniform, I volunteered to go over and do medical work in Finland. The story is complicated and, in retrospect, full of the improbable. The organization was phoney, and the plan futile. I arrived in Finland with about two hundred others to join the advance guard of about a hundred, forming the nucleus of the British Contingent of the International Volunteer Brigade. The date of our arrival was 13th March, 1940, the day after the Russo-Finnish armistice was signed. There was a possibility, or so many of us thought, of further fighting and a spread of hostilities to Scandinavia, and so we stayed. I liked the Finns, and made many friends.

It seems odd now to think that Britain tried to go to war with Russia and Germany at the same time ! It took ten months to get out of Finland and several attempts failed. Meanwhile, Norway fell, Belgium and Holland were invaded, and the collapse of France was followed by the astonishing evacuation of Dunkirk. Daily and anxiously we listened to the disastrous news. Some of the Finns were so anti-Russian as to be pro-German ; and some, particularly the Swedish element, were pro-German by association. They and all the others expected England to ask for terms.

I was lucky, and in December, 1940, through a contact with Tanner, the Finnish Minister of Finance, and Möller, the leader of the Swedish Social Democrat Party, I had a personal interview with the famous Madame Kolontay. She had been Soviet Minister in Stockholm almost since the Revolution. Once a noted aristocratic beauty, and a friend of Stalin, Madame Kolontay ruled her associates more like a queen than a comrade, and I was much impressed by her presence and ability. Others had had to wait six months or more, but my visa came through in four days. I was a little disappointed, because it meant leaving Stockholm at once, and I had just made arrangements to see all the chief hospitals in the area. I returned to Helsinki, but had to wait three weeks for a visa to go through Syria; but in the meanwhile snow arrived and I was able to ski. On January 4th, 1941, I started my journey, via Leningrad, to Odessa, Constantinople, Ankara, Beyrut, Haifa and Cairo.

It was most interesting to have a glimpse of Russia. A few hours loose in Leningrad, without permission, showed me a poverty-stricken city and a population nervous of contact with a stranger. In the train I was lucky to be alone in a compartment with a mining engineer and his mother. He was evidently a senior official, and spoke a little German. We became quite friendly in the course of the 48-hour journey, but he would talk only if the train was in motion, the corridor door closed, and he spoke quietly, leaning forward in his seat and watching the door. Before the train stopped in Odessa he said he would like to say good-bye, because in Odessa we must not know each other. We parted most cordially, and on the platform passed as though we had never met.

In Odessa there was an organized tour from the official travellers' hotel, where I stayed with half-a-dozen assorted travellers, including two King's Messengers from the Embassy in Moscow. We saw the outside of many fine buildings, including the city hospital. I asked the guide, a charming girl who spoke perfect English, if I might see the inside of the hospital. She said "No," and when I asked why, she looked embarrassed, and finally said, "But why do you ask? Would you do this in another country? Doctors there would not want to show you their work!" She was incredulous when I assured her that in all countries doctors were delighted to show their hospitals to visitors from abroad. She promised to inquire, and later told me that unfortunately it was not possible for me to see the hospital. I did, however, see a performance of "*Esmeralda*" at the Opera, and that was a wonderful experience. The Russians are proud of their Opera.

It may well be several generations before the Russian standards, as a whole, come anywhere near the level of developed Western countries. Meanwhile, many are in a very primitive state, and everywhere very sensitive—a sensitivity stimulated by virulent propaganda and much distorted information.

The people may not know what they miss, and outsiders may not know what the Russians lack. They are taught that there are many evil and scheming enemies ready to attack Russia, and freedom of speech is unknown in a rigid Police State with iron discipline.

On reaching Egypt on 19th January, 1941, I went to the Embassy with an introduction from the British Minister in Finland to the Ambassador, but having lost all count of time, called on a Sunday, when of course there was no one there. However, two days later I had my commission in the R.A.M.C. and was posted to the 63rd General Hospital at Helmieh. The first time I wore service dress is vivid, for I got mixed up with the belt and walked down to the Mess with the shoulder strap back to front and over my left shoulder.

Five weeks later, the O.C. Medical Division was posted to take No. 24 Casualty Clearing Station to Greece, and I persuaded him to take me. We sailed in convoy on 9th March, 1941. On the voyage I remember an officer of the 4th Hussars asking if anyone knew the difference between going to France and going to Greece. The answer he provided was that, in the first case, no one expected Dunkirk and it was sucessfully evacuated. In the case of Greece everyone expected it to be evacuated and did not think it would be successful. For me this was a new angle. I was guileless and optimistic. I had a tennis racquet with me and all my kit.

Greece in Spring was very lovely. We spent a week at Kyphissia near Athens, and on a glorious afternoon, after a fall of snow on a wild night, I climbed Pentelicon and looked over Boeotia to Marathon and on the other side across the bay of Salamis. The sun shone and snowdrops and crocuses were bursting through the snow.

From here we moved near Larissa and waited for our equipment. There was no sense of urgency and the weather was perfect. In the distance was snow-covered Olympus, and in the foreground rolling downs, fallow and green shooting corn. Large flocks of sheep grazed quietly under the guardianship of huge and very fierce dogs. Then suddenly came a warning to expect paratroops. Everyone became security minded and passwords and emergency plans were the order of the day. A C.M.P. corporal came to test our security. We tested his and found he had no identity card and had forgotten the password. He was much abashed, but managed to get someone to prove his *bona fides*.

Shortly afterwards came news of the German break-through in the Monastir gap. Orders and counter orders came in a flood, and the Sub-Area controlling the district, to which I had just been attached as "dog's-body," or unofficial D.A.D.M.S., moved back. No. 24 Casualty Clearing Station and a Company of No. 189 Field Ambulance remained. I asked if I might stay and do liaison work between them and ambulance railhead about thirty miles away. The next three weeks, as we moved back and confusion grew worse and worse, were spent almost entirely on a motor-cycle. Contact with formation H.Q. was soon lost and I enjoyed using the authority of the A.D.M.S. It was impossible to get a clear picture from anyone and units hid so successfully that it was very difficult to find them. The roads were vile and the German planes numerous, traffic was forced off the roads in daylight, but a motor-cycle was fairly inconspicuous. One could usually tell if there were planes about by the behaviour of the adjacent Greeks. They were as sensitive as magpies in a wood. Except once or twice I got warning. Drivers who had to be on the road seemed to drive as fast as they could between points. If they were attacked, this meant that they swerved off the road and crashed because they were unable to pull up. I thought it better to go slow if I thought there was anything near, so that it was possible to pull up quickly and make a dash for cover.

At night the roads, which were nearly all on embankments, were jammed and the number of vehicles upset into the fields was astonishing. On the whole, ambulances were respected.

At one ambulance railhead, Thebes, there were some unpleasant moments. A hospital train was in a small station crowded with rolling stock. Early in the morning it was loaded, and then the Germans started an intensive *strafe* of the whole area, including the station. Most of the glass in the train was shattered and woodwork splintered. The quiet courage of the wounded, who could do nothing to help themselves, was something never to be forgotten. Three out of four locomotives available were damaged, and several lines blocked. Attacks were frequent throughout the day. Urgent messages were sent for ambulances to evacuate the train, and in the interval I found occupation in trying to get the remaining locomotive up to clear the train out of the station. The Greek drivers went off and I chased them with a rifle. In case of accidents I got them to show me how to drive and did some of the shunting myself, which was rather entertaining. So engrossed was I in shunting and using gradients to roll wagons out of the way that when I came back to say the line was clear the train was almost evacuated. The attacks were unsettling, and once I was standing by the engine just outside the station when a Messerschmidt made a low level sweep and came straight for it. I dropped flat, but the plane shot over the engine without firing. I looked up to see the engine-driver whom I had recently chased looking out of the cab and grinning at me.

Finally, in the afternoon we drove the train out of the station. It was essential to get it away as there were only two or three ambulance trains in Greece. For a short while before leaving there had been a lull, but when just clear of the station the *strafe* started again and a bomb hit the platform where the train had been. The train was very clearly marked with red crosses, but was in a target area. General experience in Greece was that the Germans respected the Red Cross.

When I finally reached Athens the general evacuation was beginning. I was asked by the D.D.M.S., Brigadier Large, if I

could drive a lorry. He said there were no drivers, there might be fighting in the Peloponnese, and medical stores were urgently required. I was given a three-tonner, which I took out to the central medical store with instructions to take what I wanted. There was no one available to load so I enlisted the services of stray Greeks who were rewarded with blankets and primuses. I started off in the afternoon, and watched Germans dive-bombing shipping along the coast near Athens. Luckily I got to Argos before the night jam began.

In Argos I was met by the local police, who asked if I was a British Officer. They said there was someone on the phone with an urgent message from Athens which could only be given to a British Officer. The message was, I found, an urgent one from the C.-in-C. Mediterranean, concerning a change in plan of evacuation for the night. Could I, the speaker said, find a senior officer to whom it could be delivered ? The request seemed odd, because there was supposed to be a senior officer in the neighbourhood in charge of beach arrangements. No one seemed to know his whereabouts, and he certainly had no one in touch with the 'phone. I stood in the road flashing a torch and flushed no less than three brigadiers from the head of a convoy just arriving. One of them took the message, and I stood in the background while they discussed the merits of drinks and laughed about their complete lack of knowledge of what was happening. The apparent lack of contact between Athens and Argos, evacuation centres, seemed very strange.

A *recce* party was just setting out to go further down the Peloponnese, and I followed them all night in my lorry. At daybreak we were near Tripoli, and I fell asleep at the wheel and was lucky to avoid a smash. An Australian from the *recce* party whom I got to drive, wrecked the lorry within ten minutes, and then hurried off to join the beach party. I sat by the lorry but could not keep awake. Each time I jerked awake I found the peasants had looted more from the lorry. Afterwards someone said that they only refrained from taking more because they liked the British. I pitied their enemies. Later I reckoned that in seventeen days I averaged less than four hours sleep in twenty-four. There was no transport within miles, and it was a lucky chance for me that the Military Attache from Athens, Colonel Jasper Blunt, was passing. He helped to salvage essential stores, and asked me to join his small party to be evacuated by caique from Monemvasia. After a few hours sleep and a shave I took my motor-cycle, rescued from the lorry, and went down to Kalamata to see what the position was there. I could find no one in authority and the organisation appeared to be very ineffective. The *recce* party which I had followed in the night consisted mainly of Signals, and I had given them some Field. Ambulance panniers to take on in case I could not get through. I found them in an olive grove with an Australian ambulance which they were using for transport. There seemed to be no useful purpose in remaining at Kalamata ; the area was large and it was difficult to find anyone in authority. I retrieved some of the panniers and the ambulance, and in the morning came back to Tripoli, to see how the bulk of the stores could best be used. As I left Kalamata many lorries were arriving crammed with Australians who had been travelling all night, and all along the road were large numbers of Yugo-Slavs who had fought at Monastir. They were hoping to be evacuated from Kalamata. One old veteran, an officer, who spoke French, said : " In the last war the British always gave us lifts, now we have to fend for ourselves and it is a case of devil take the hind-most." He looked worn and sad. He and all the British troops were captured at Kalamata, owing to the infiltration of a small detachment of German troops who upset evacuation plans. Their infiltration was possible chiefly because of bad organisation which allowed them to drive " straight up the road." There appeared to be no defences.

Arriving in Tripoli I learned that evacuation was to be speeded up, as the Germans were approaching from Patras. The New Zealanders were providing the rear-guard, and were really first-class troops. They had a medical officer, Fisher, and plenty of stores. I therefore left the ambulance and most of the stores with the local Greek hospital, where there were some badly wounded British casualties. After dark our party left Tripoli, and we were joined by several officers, among them the man who had sent the urgent message from Athens to Argos. Next day we spent at Monemvasia listening to the sound of distant bombing and the constant drone of German planes exploring the neighbourhood, flying low and machine-gunning at intervals to get troops to give away positions. The bombing, we learned later, signalled the sinking of a crowded evacuation ship and two destroyers, the *Wryneck* and *Diamond*, which attempted rescue work. Owing to ceaseless escort duty they had run out of ammunition, and the ship had taken too long to load and was caught near the coast in daylight.

In the afternoon I was eating some bread and salmon in a hut when the door opened and an elderly officer walked in. All I saw was a single pip and " something." Out of courtesy I stood up and asked what I could do. In a gruff voice the visitor said " I am General Freyburg. I am looking for . . . " I was intrigued to see what a general looked like and made a mental note to recognise the insignia of rank the next time I saw them. At dusk we were relieved to hear the chug-chug of the caique, and after dark the whole party, about fifteen, including Colonel Quilliam, the D.M.I. Greece, went on board. Some of my precious panniers were also loaded as we were making for Crete via Kythera. There was a fresh breeze and a short choppy sea, overhead brilliant stars with dark banks of cloud. Early next morning, 28th April, we reached the little harbour on Kythera where we were going to lie-up for the day.

During the morning there were several visits from German planes. An ancient British Walrus plane came over and was attacked by two Dorniers. It was an amazing sight, the two Dorniers were swooping from all directions, and the old Walrus dipped and side-slipped nearly at sea level until gradually they faded away in the distance. Later I heard that the Walrus escaped: a masterly performance.

In the afternoon, which was brilliantly clear, I climbed with another to a small monastery on the highest point of the island, about two thousand feet up with a wonderful view. The water supply of the monastery was a large rainwater roof tank, beautifully fresh and ice cold. It had for years been deserted except by shepherds. When we returned we found a message had been dropped by a seaplane to say that a cruiser was coming in the evening to take off 750 R.A.F. men. We had not known they were on the Island, but contact was made through the locals, who found them on the other side. It was decided to leave the caique to go to Crete with two Greeks, and after dark, when the cruiser *Auckland* arrived, we got aboard and were able to direct them to the R.A.F. Next morning we arrived at Suda Bay in Crete where we saw the mast-tops and wrecks of many ships. The R.A.F. were disembarked, but Colonel Quilliam asked me to go on with him in the cruiser to Alexandria. It had been an interesting introduction to the Army, and good fortune had given me a useful job and opportunity for independent action.

The next two years were spent in the Western Desert, first with 7 M.A.C. and then with 16 M.A.C., backward and forward in the various desert "stakes." It was curious to note how many began to think that it was almost natural and inevitable that first we should advance and then the Germans, then another British advance.

The advance in November, 1941, I shall always remember vividly because of the number of tanks we lost. Wavell has recorded the battle in his despatches as one of the few occasions in history when an army has advanced against an enemy with almost equal force and in some respects better guns. The German 88 mm. was deadly. The desert was littered with our tanks singly and in groups of half-a-dozen or more. Each group we met, we thought: "Now these must be German," but they never were, partly because the German recovery system, even in retreat, was so good. The gallantry of our armoured troops was beyond praise.

Later came the battle which led to the loss of Tobruk and the retreat to Alamein. I had command of No. 16 Motor Ambulance Convoy, and had a strenuous time trying to keep contact with constantly moving medical centres over a trackless desert. Locations were map references in space without mark, and it was all map and compass work. At the beginning of the battle hopes were high, and I remember at Corps H.Q. they were most optimistic. The German tank losses were reported to be very great and the R.A.F. pounded them in the " cauldron " : victory was in sight ! Then came the disastrous day when we lost the bulk of our armour and the retreat began, but few expected to go back to Alamein, and all thought Tobruk was secure.

We were in the vicinity of Tobruk when one morning an Auster plane landed nearby, and I asked the pilot over for a drink. He said that orders to *recce* a landing strip for Kittihawks had just been cancelled, and air-cover for Tobruk withdrawn, which meant Tobruk had fallen. This was the first news we had, and when I passed it on to the A.D.M.S. 7th Armoured Division he was incredulous. No one thought it was possible, everyone had been thinking in terms of the relief of Tobruk at an early date. Later, stragglers from Tobruk who had broken out came into the camp and we heard more of the story of confusion and unpreparedness. Some of them were very bitter, and bewildered by the way things had happened.

Subsequently, in Cairo, I heard that on the night of 12th June they were ready to announce a great victory, and on the 13th June had instead to accept retreat.

Back at Alamein there was fluctuating fighting, and my car went over a recently-laid German mine. This sent me out of the desert for a while, and I missed the great advance. In an effort to follow up I got myself posted from No. 15 (Scottish) General Hospital to No. 99 Sub-Area, which I heard was bound for Tripoli non-stop. Instead we went to Alamein to do salvage work, suffocated by swarms of flies. It was a bitter disappointment. In April, 1943, I was posted second in command No. 200 Field Ambulance. In due course we landed in Sicily with No. 231 (Malta) Brigade in an independent Brigade Group. A finer set than the " Malta " Brigade I never hope to meet. My company was with the Brigade while the rest of the Field Ambulance were working for Corps, so we had a gloriously independent time. The Brigadier (now General Urquhart of the 1st Airborne Division), was worth a brigade by himself. How he survived is a miracle. The difficulty was to keep up with him. The brigade casualties were about the highest in Sicily, but they had done a grand job.

After the fighting was over we spent a wonderful fortnight in a baronial mansion at Piedmonte with a glorious view of Etna and over vineyards leading down to the sea, with sunshine and grapes abounding and much good wine.

Next came an independent landing in Italy at Porto Venere, beyond troops advancing from Reggio. Plans were a bit mixed as the landing point had been changed three times in almost as many days. Ultimately, instead of landing before dawn, much remained afloat when daylight came. Steep hills overlooked the little harbour of Porto Venere, and the Germans played havoc. By nightfall there were about 300 casualties and the position was not bright. The casualty centre had been bombed and mortared and so moved to a railway tunnel one-and-a-half miles long, the other end of which was later in enemy hands. A slight gloom was dispelled by the sudden news of a landing at Salerno, the Italian collapse, and the arrival of *recce* units of the 5th British Infantry Division which had pushed ahead full speed to our assistance. Next morning tank craft took off the wounded, and we worked peacefully in the sunshine looking at olive groves and a placid bay. It was hard to reconcile with the previous day. All through the war it was almost unreal how suddenly death came and vanished, leaving birds singing and the country beautiful.

A month later the Brigade was withdrawn to Sicily and we spent a glorious month in Taormina, the beauty spot of Sicily, waiting for a boat to England. The voyage was uneventful except for a few alarms. We heard the escorts sank two submarines. In the Irish Sea, the escort flushed a submarine with depth charges and fired at it. The submarine replied indignantly, and there was a brisk exchange before they realised it was British. We were a day ahead of schedule and the submarine had had no warning. We arrived at Avonmouth in November, 1943, and were sent straight on to near Ipswich. In mid-March we went to the invasion concentration area in the New Forest. The Brigade had been six years in the Middle East, three hard years of which were in Malta. They were now put in thick woods, eight men to a leaky bell-tent, no floor-boards and much rain ; there was ice on the water and many were flooded out. There were no recreation tents, nor drying tents. At first we were confined to camp with security plus plus. Not unnaturally, the number " absent without leave " was phenomenal, but they all came back for the fighting.

Two days after I had been briefed with the C.O. and seen models and plans of the invasion point, and we were getting " all set," a sudden posting came to take command of No. 163 Field Ambulance. The translation to Hampstead in comfortable billets out of the tension was unbelievable. The 231 Brigade had joined 50th Division and landed in the assault wave in Normandy. Their casualties then and after were very heavy. We landed a month later, and joined 30th Corps. We had a forward role from then until after the 1st Airborne landing at Arnhem. The rapid advance through Belgium and Holland and up to the Rhine at Arnhem was fascinating. The entry into some of the towns was unforgettable. The tanks were mobbed by hysterically happy crowds.

My unit went with the medical support accompanying the armoured striking force to join the two American Airborne Divisions dropped between the Escaut Canal and Nijmegen. The aim was to relieve the 1st Airborne Division fighting at Arnhem. Unfortunately, this was not possible, and reports were received of large numbers of wounded in need of medical supplies and assistance.

The 1st Airborne reserve medical supplies were given to No. 163 Field Ambulance, with instructions to go forward and see what could be done to give aid. Attempts to cross the river were unsuccessful, and so on 24th September I called for four volunteers and with Captain Lewis of the 1st Airborne, who was unfortunately killed that night, went down to the river under cover of the Red Cross. We found an abandoned assault boat and took over some supplies. I left the party by the bank and went forward to reconnoitre. There was a lot of noise, but it was impossible to tell who was firing at whom. The exact location of the 1st Airborne troops was uncertain and, not unexpectedly, I entered the German lines. I demanded to see the senior officer in order to request passage or, if refused, the safe return of my party. I was blindfolded and for about an hour was led from place to place to find an officer sufficiently senior to make a decision. Ultimately they refused, but said my party would be returned, while I would be held for the night. Later in the evening they said I would be regarded as a prisoner, but I took a very strong line and, ultimately, it was decided that next day I should go to Apeldoorn and discuss matters with the senior medical officer in the region. Unknown to me, the A.D.M.S. of the 1st Airborne Division, Colonel Wanack, had that day approached the Germans to request evacuation of the wounded.

Next morning I met the German A.D.M.S. and it was arranged to establish a British hospital at Apeldoorn, staffed by Airborne medical personnel, many of whom had already been captured. About two thousand casualties passed through this centre. They came in exhausted and battered by eight days of ceaseless fighting, short of food and water.

Many had been re-wounded and many killed in the casualty stations. Yet their high morale and invincible cheerfulness was almost unbelievable. It was not possible to think of them as prisoners. At Apeldoorn a large barracks was allocated for the hospital, and the first night with negligible equipment and very few medical personnel we had to cope with over seven hundred, chiefly walking wounded. At 5 a.m. the next morning a German officer came to say that five hundred were to be detailed for evacuation by train at 8 a.m. to make room for the further large numbers expected. It was necessary to go round and wake the wounded from what was in many cases their first sleep for many days, and yet there was no murmur of complaint from any of them and they went off singing.

A large number of Airborne medical personnel, with Colonel Wanack in charge, organized the hospital, and it was a privilege to be with them. They were dead tired, yet indefatigable and cheerful.

I acted as intermediary with the Germans and Colonel Wanack asked me to be his second in command. There were several thousand German wounded in the area and we emphasized that British troops might break through at any moment. It was in their own interest, therefore, to treat our wounded well. This attitude was successful, but later the Germans recovered confidence, and many wounded and medical personnel were evacuated to Germany, and it was evident that all movable wounded would follow. I therefore decided to try and return to the British lines, taking a list of the casualties which had been compiled in the hospital. On a particularly wet and dark night I climbed out of a window and slid down an improvised rope of blankets with one of the Padres, Dan McGowan. We crawled past the sentries, and it was so dark that we nearly bumped into one or two of them. Luckily they were preoccupied by desire to protect themselves from the rain and wind.

After a three-day trek, travelling by night, using map and compass, and hiding by day, we arrived at the Lower Rhine at the point at which we had decided to cross. Unfortunately, near the bank, where we had to keep very quiet and crawl, we became separated. As it was almost dawn, I decided to swim across, expecting McGowan to follow. He did not come and I learnt later that he was recaptured.

On the far bank I found the 101st American Airborne troops, whom I was glad to meet. They marched me off to establish bona fides and, after a sleep, passed me through to No. 30 Corps H.Q., where I was able to deliver the list and give news. It was now 19th October and, in the interval, fighting had settled down for the winter in front of Arnhem, while my unit had been withdrawn to Antwerp. I learned that the Germans had kept the supplies, but sent back the men who had crossed the river with me.

In Antwerp it was at first pleasant, but when the buzz-bombs and rockets came in numbers the place " went dead " and was rather depressing. Three times we prepared to move and three times the place we were going to was damaged. I kept the men working one Saturday afternoon preparing to move and the cinema where most of them had intended to go got a direct hit ; we were lucky. My batman and driver for many weeks kept making remarks about their miraculous escape.

Then came the Rhine crossing and we were moved up to work in Belsen Camp which we reached ten days after it was opened. Piles of corpses lay everywhere naked and emaciated, and huge grave pits were being filled with thousands of bodies. Sub-human creatures prowled about apathetically, and the huts were so crowded that it was impossible to tell the living from the dead. No. 32 Casualty Clearing Station and No. 11 Light Field Ambulance had done magnificent work, and the way my fellows settled down to help was grand. I was very proud of them. Finally, two General Hospitals arrived and we moved on towards Luneberg to take over another " horror camp " at Neuengamma near Hamburg when it was reached by our troops.

The Germans were fighting in battle-groups at this time, and on a reconnaissance I made contact with one group and demanded entry into Neuengamma. They took me blindfold to see their commander. He assured me the camp had been evacuated, and I assured him they would be held personally responsible if we were denied entry and subsequently found anything wrong. I told them something about Belsen and they were incredulous. In a conversation with several officers, they likened Hitler to Jesus Christ and expressed the most fanatical sentiments. I returned to our lines and alternative plans were made. Later we found that the camp had been evacuated and the survivors put on a ship for Norway, deliberately with the intention of getting them sunk by allied planes, and they were, in fact, bombed and set on fire. Some survived and were landed at Lubeck. I got in to Lubeck on the morning of the German disintegration, and saw the surrender of a German army with units breaking up into rabble crowds. The P.O.W. areas were a sight never to forget. The German forces had been at their last gasp.

On 1st June, 1945, we moved from the North to Goslar in the Harz mountains and took over an area nearly a hundred miles square from the Americans. My unit had to try and arrange feeding and supervision for hospitals containing nearly 40,000 German casualties, and it was a busy time. Later we moved to Hanover and from there in September, 1945, I was posted A.D.M.S. to the 5th British Infantry Division in Brunswick. The war was over and I found time to get some ski-ing in the Harz and plenty of riding. I was sorry to leave, but felt it was time to get back to civilian life and prepare for the future.

This article has proved to be much longer than originally intended. So many scenes spring to mind and when once started it is difficult to know what to include and where to stop. I was asked to recount personal experiences. If the recurrent personal pronoun is wearisome, I can only beg the reader's indulgence for what is an unaccustomed task.

